# Harnessing Grape Pomace, a Multifunctional By-Product from the Wine Industry for High-Value Salad Dressings

**DOI:** 10.3390/molecules30030693

**Published:** 2025-02-05

**Authors:** Luciano Mangiapelo, Nicola Pinna, Francesca Blasi, Federica Ianni, Giuseppa Verducci, Lina Cossignani

**Affiliations:** Department of Pharmaceutical Sciences, University of Perugia, 06126 Perugia, Italy; luciano.mangiapelo@dottorandi.unipg.it (L.M.); nicola.pinna@dottorandi.unipg.it (N.P.); francesca.blasi@unipg.it (F.B.); federica.ianni@unipg.it (F.I.); giuseppa.verducci@unipg.it (G.V.)

**Keywords:** functional foods, fruit waste, antioxidant properties, phenolic compounds, HPLC, sensory analysis

## Abstract

Grape pomace (GP) has gained attention for its potential to be valorized into functional foods due to its rich composition of bioactive compounds. In this work, GP has been exploited to develop plant-based mayonnaise alternatives and salad dressings. The influence of the water-to-oil ratio, percentage content of GP, lecithin, and vinegar on the viscosity and physical stability of the obtained emulsions have been investigated by the Design of Experiments. Two formulations, one high-oil (70%) and the second high-water (60%), were further studied for their potential applications. The selected samples were subjected to an accelerated stability test (60 °C for 21 days) to verify the influence of GP on oxidation protection. The high-water sample, combined with 8% GP, showed lower primary (peroxide value < 20 mEq O_2_/kg fat) and secondary oxidation (anisidine value < 55) parameters than the high-oil sample, highlighting the GP antioxidant activity. The phenolic profile of all samples by HPLC was also determined. Lastly, a sensory analysis was conducted, showing the highest overall acceptability for the oil-rich sample. The obtained results contribute to highlighting the potentiality of GP in the formulation of healthy foods, adopting the zero-waste approach for the full exploitation of this underutilized resource.

## 1. Introduction

Nowadays, consumers are aware of the significant influence of diet on their health, and the demand for nutritious and healthier food is increasing. Among oily dressings, mayonnaise is not generally considered a healthy salad dressing option due to its high cholesterol and fat content [1]. For this reason, there has been a growing trend toward replacing eggs with plant-based ingredients that can provide thickening, stabilizing, and gelatinizing properties, as well as a better nutritional value than egg-based mayonnaise. Most of these ingredients are based on polysaccharides, such as xanthan and guar gum, and vegetable proteins, which provide important rheological characteristics in the production of seasonings and emulsions [2]. Using these components allows us to develop products with a lower oil-to-water ratio, and with the advantage of disposing low-fat and low-calories mayonnaise alternatives. The current trend towards sustainable development aims to valorize food waste and by-products as efficient strategies to exploit the benefits of available resources and improve food security, sustainability, and circularity in food systems [3].

Among agri-food waste and by-products, grape pomace (GP) stands out for its huge amounts generated every year worldwide, especially in Italy, France, Spain, and California [4]. Recent studies highlight the significant potential of GP as an innovative ingredient for the enrichment of feed and food [5], thanks to its high content of bioactive compounds, including polyphenols, dietary fiber, fatty acids, and other nutrients, such as minerals (K, Fe, Zn, and Cu) and vitamins (C, group B, and E) [6].

The polyphenolic profile includes flavonoids (anthocyanins, flavonols, flavanols, and proanthocyanidins), phenolic acids (e.g., gallic acid, caffeic acid), and stilbenes (resveratrol) [6,7]. The composition varies depending on factors such as grape variety, vinification process, and climatic conditions. For instance, Onache and coworkers investigated the phenolic content in different white and red grape pomaces. They concluded that white grape pomaces were characterized by the presence of tannins, (+)-catechin, and (-)-epicatechin, while red grape pomaces were rich in anthocyanins, quercetin, syringic acid, and pinostrobin [8]. The bioactivity of GP phenolic compounds has been extensively investigated, and numerous healthy properties have been reported [6,7]. Among these, antioxidant activity prevents chronic diseases such as cardiovascular and neurodegenerative disorders. Moreover, GP phenols exert anti-inflammatory, anti-cancer, and anti-diabetic activity [6,7,9]. Dietary fiber, the more represented fraction in GP, is also valuable for technological and healthy properties. GP contains substantial amounts of insoluble dietary fiber (16.44–63.70 g/100 g: cellulose, hemicellulose, and lignin) and smaller amounts of soluble fiber (0.72–12.78 g/100 g: xilan, glucan, and galactan) [10]. The fibers are essential for gut health and have been linked to improved digestion and cholesterol regulation [5,6,7]. The bioactive-rich composition of GP makes it a valuable ingredient to increase the nutritional and healthy properties of feed [10] and food [5,11,12]. GP-derived extracts and powders have been successfully incorporated into diverse food matrices, including baked goods, meat products, beverages, and dairy alternatives [13]. These additions not only increase the nutritional profile of foods, but also improve their sensory properties and shelf life thanks to the antioxidant activity of GP. Although GP has been shown to improve the functional and healthy properties of mayonnaise and dressings [1,2,14], sensory acceptability is key. Researchers are working on optimizing extraction methods and processing techniques to balance taste, texture, and health benefits, ensuring consumer satisfaction.

The present paper aims to investigate the potentiality of GP in the formulation of healthy mayonnaise-like products, assessing the GP impact on emulsion properties and antioxidant activity. The zero-waste approach was followed; this allows the exploitation of all of the bioactive components of GP and reduces processing costs, fully satisfying the sustainability criteria. An approach based on the Design of Experiments has been used to verify the influence of the composition of GP-based formulations on their physical stability. In particular, the water-to-oil ratio and the % GP content are relevant variables influencing the nutritional and healthy properties of the investigated products. An accelerated stability test was conducted to verify the antioxidant protection provided by GP phenolic compounds. The inclusion of GP in the proposed dressing enhances its antioxidant capacity and adds dietary fiber, offering a functional food solution that aligns with health-conscious consumer demands.

## 2. Results and Discussion

### 2.1. Optimization of GP-Based Dressing Formulation

In the present research, the development of innovative dressing containing GP was carried out by the Design of Experiment (DOE), a statistical technique which allows us to investigate the impact of multiple different factors on an experimental process. This technique enables researchers to reduce the number of experiments they need to perform by recommending the best operating conditions and combination of parameters for their process.

MODDE 5.0™ software was used to evaluate the influence of formulation composition on the viscosity and syneresis of salad dressing with added GP. The four variables selected as factors are the contents of the formulation components; that is, the % water content and the relative % oil content, the % content of GP, lecithin, and vinegar. The range selected for the water-to-oil ratio, from 30:70 to 60:40, was chosen to test mayonnaise-like formulations, as well as dressing with lower lipid content, to meet the needs of consumers who prefer products lower in fat and calories. Table 1 shows the results of the selected responses, viscosity, and syneresis. It is possible to observe that N5 and N7 samples, both having high oil content, had the highest viscosity and the lowest syneresis value. Moreover, N10 and N12, among the samples with high water content, showed the highest syneresis, indicative of low physical stability.

**Figure 1:**
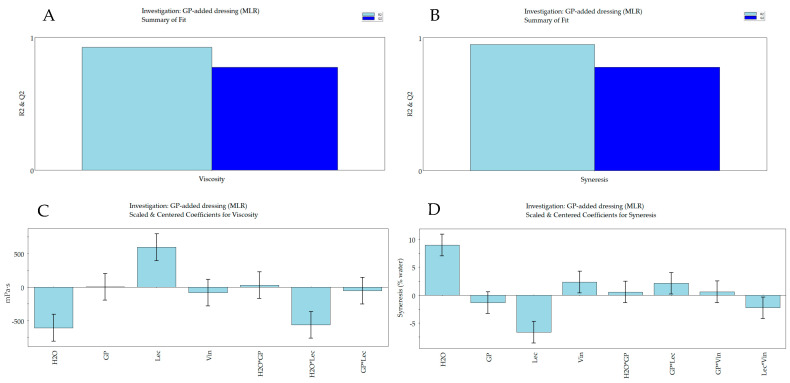
Statistic of the model—R^2^ (goodness of fit) and Q^2^ (goodness of predictability) values for the response viscosity (**A**) and syneresis (**B**). Coefficient plots showing the effect of percentage of water (H_2_O), grape pomace (GP), lecithin (Lec), vinegar (Vin) on the following responses: viscosity (**C**) and syneresis (**D**). MLR, multiple linear regression; * interactions between two variables.

Figure 1A,B shows the statistical parameters, R^2^ (which describes how well the model fits the experimental data) and Q^2^ (which describes how well the model will predict new data), indicating the quality of the mathematical model, obtained for the response viscosity and syneresis, respectively. The results show the validity of the developed models, and therefore their usefulness for optimization and prediction, with R^2^ values ranging from 0.925 for viscosity to 0.950 for syneresis, and Q^2^ values from 0.775 for viscosity to 0.778 for syneresis. In Figures S1 and S2, the response prediction plots of the four investigated factors on viscosity and syneresis are reported. The same figures show the relative surface contour plots generated by the software as a function of two selected variables (water and GP percentages), keeping lecithin and vinegar content at the constant value of the center point. The influence of the selected variables on the responses is readily evident from the coefficients of each factor in the mathematical equation, shown in Figure 1C,D, for the response viscosity and syneresis, respectively. Furthermore, it is possible to observe an opposite influence of water % on the two responses.

In fact, the considered factor was inversely correlated with viscosity, and directly correlated with syneresis. The viscosity of the GP-added formulation was higher, while the syneresis was lower, in the samples with lower water content. Lecithin content was also found to be a relevant factor, showing an inverse correlation between the two responses, but the trend was opposite, as the higher lecithin percentage corresponded to the higher viscosity and the lower syneresis. The coefficients for the vinegar variable show a positive influence on syneresis, even if it is lower than water % and lecithin, and an even lower negative impact on viscosity. The above-reported opposite influence of the variables on the two responses was predictable, as a more viscous sample has a higher physical stability, i.e., a lower syneresis. Regarding the influence of the % content of GP, it turned out to be almost irrelevant for viscosity, but it had a slightly negative influence on syneresis. The dietary fiber composition of GP, with a high insoluble-to-soluble ratio, may provide an explanation for the result obtained for the viscosity [15]. It has been reported that the interactions between cellulose and different polyphenolic compounds can improve the physical and oxidative stability of lipids in O/W emulsions [16]. Moreover, Su and coworkers have proposed plant-based ingredients as replacers for egg-based mayonnaise. In particular, they proposed protein-polysaccharide-lecithin composite particles to formulate emulsions with high physical stability [17]. The presence of proteins and polysaccharides in GP can help to increase the emulsion stability and explain the observed reduction of the syneresis in the investigated products.

There is plenty of evidence of the effect of dietary fiber from fruit and vegetable by-products on solubility, viscosity, hydration property, oil-binding capacity, and antioxidant activity in food products [13]. Regarding creamy food products, it should be emphasized that GP has been added more to dairy products [18,19,20,21,22,23,24,25] than to salad emulsions and mayonnaise [14,16,26]. In this regard, Diaz-Ramirez and coworkers have proposed the integral valorization of GP for antioxidant Pickering emulsions, but they used bacterial cellulose produced from GP as the raw material, and a polyphenolic extract obtained from GP [16]. The authors found that the synergistic effects of nanocellulose and GP phenolic extract resulted in promising innovative emulsions with prolonged physical and oxidative stability. Many sources in the literature refer instead to the exploitation of apple pomace (AP), as a multifunctional by-product from apple juice production, for the development of vegan mayonnaises and salad dressings. Pickering emulsions stabilized by AP particles were prepared as novel cholesterol-free mayonnaise, which showed long-term storage stability [27]. The stability was mainly attributed to the irreversible adsorption of AP particles at the interface of oil droplets and to the network structure limiting the free movement of droplets. In another study, the influence of composition parameters on the viscosity of AP-enriched mayonnaise was investigated and, differently from the results of the present study, the water percentage and AP addition had a positive influence on the viscosity of the formulates [28]. It should be underlined that a lower insoluble-to-soluble fiber ratio characterizes AP with respect to GP [27]. New pectin-containing apple nanofibrils were prepared and tested for the emulsifying properties [29]. The emulsion microstructure showed that the fibrils were irreversibly adsorbed at the oil–water interface, providing electrostatic stability. Recently, Hollestelle and coworkers have investigated the co-stabilization mechanism of solid particles and soluble compounds in hybrid Pickering emulsions stabilized by AP powder [30].

The nineteen prepared samples were further characterized for the emulsion stability and pH value, by measuring the oil percentage separated after centrifugation. The results, reported in Table S1, show a higher oil separation for N3 and N15 samples, due not only to the high oil content, but also to a combination of the other considered variables. The pH value was higher for N7 and N11 samples, characterized by lower water content and a higher GP addition.

Lastly, the color parameters were determined for N1–N19 samples, as the color represents the important factor affecting consumers’ acceptance and selection of food. The results, reported in Table 2, show significant differences between the samples, with the predominant role of the anthocyanin-containing GP influencing the color of formulations. In general, lightness (L*) and blueness values (b*) were always lower than a commercial mayonnaise sample, while, as expected, redness value (a*) was always higher. Regarding L* and b*, the lower values were obtained for the samples with a higher GP addition (8%) and lower water content (30%). These same conditions, together with the low lecithin content (1%), corresponded to the highest a* value, recorded for N3 and N15 samples. Table S2 shows the differences between the color parameters of the GP-added formulation and the commercial mayonnaise. It is possible to observe that ΔE*ab, indicating the color changes perceptible to the human eye, were more evident for N3 and N13 samples, characterized by a high GP addition and low water content. Similar results have been obtained by other researchers, who have studied, among other parameters, the color of GP-fortified salad dressing [14] and AP-added dairy products [28,29]. It is evident that the addition of GP has a strong impact on the product’s color; therefore, it is essential to verify its acceptability by panelists and consumers. Color changes might affect product appeal. In particular, some consumers might be sensitive to changes in the color of a food product (i.e., mayonnaise substitute), which is typically pale, thus changing their expectations and sensory perception (taste/flavor).

### 2.2. Accelerated Stability Test

**Figure 2:**
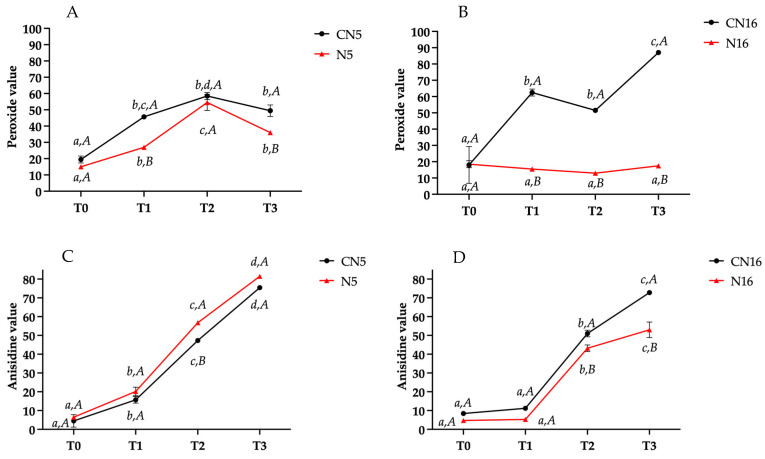
Values of peroxides (**A**,**B**) and anisidine (**C**,**D**) for GP-added samples, N5 and N16, respectively, and the relative controls (CN5, CN16) at different storage times (T0, T1, T2, T3). Different uppercase letters indicate significant differences (*p* < 0.05) between the added and control sample at each time; different lowercase letters indicate significant differences (*p* < 0.05) between different times for each added and control sample. T0, immediately after their production; T1, after 7 days at 60 °C; T2, after 14 days at 60 °C; T3, after 21 days at 60 °C. N5 and N16 correspond to the samples reported in Table 1.

The accelerated stability test has been conducted to verify the progress of the oxidative processes in the selected N5 and N16 formulates. Control samples (CN5 and CN16) have been prepared for both products without adding GP. The parameters selected to monitor the oxidation progress in emulsions were peroxide value, anisidine value, Totox value, and spectrophotometric indexes. Figure 2A–D, shows the results obtained for peroxide (A–B) and anisidine value (C–D) in the GP-added samples, N5 and N16, respectively, and the relative controls (CN5, CN16), at different storage times (T0, T1, T2, T3).

Regarding peroxides, intermediates in the autoxidation/photooxidation processes, and markers of primary oxidation, Figure 2A,B clearly highlights the protective effect of GP on primary oxidation products. It is possible to observe that the values for both added samples were significantly lower than those registered for control samples, at each time of the accelerated stability test. The only non-significant difference between the control and added sample was registered for N5 at T2. The peroxide trend during the accelerated test shows, for the N5 sample, an increase until T2 and lower content at the final T3 time, with a significantly lower value for the GP-added formulation at T1 and T3 (*p* < 0.05). For the N16 sample, very low peroxide values were measured at each time, without significant differences at the different times, while a significant decrease was obtained at T2 compared to T1 for CN16. This last sample, however, was characterized by the highest peroxide value (87.0 meq O_2_/kg oil) at T3, and it is noteworthy that at this final time, the highest difference was recorded compared to the control sample (17.5 meq O_2_/kg oil). The higher antioxidant power observed for the N16 sample can be attributed to the higher GP addition (8%) compared to N5 (4%).

Less evident was the antioxidant efficacy of GP assessed by anisidine value (Figure 2C,D), a measure of secondary oxidation products, such as aldehydes and ketones. These compounds, deriving from fatty acid degradation, contribute to the unpleasant taste and odor associated with rancid oils. In particular, N5 anisidine values at different times of the accelerated test were only slightly higher than CN5, with no significant difference (*p* < 0.05) at each time, except for T2. For N16 and CN16 a similar trend was observed, with a slightly lower value for the added sample compared to the control one at each time. However, these differences were non-significant (*p* > 0.05), except for T3, for which a significantly lower anisidine value was obtained for N16 compared to CN16. These results confirm, at least for the last test time, the antioxidant protection of GP in the samples with high GP addition. The different composition of these formulates, and in particular, the higher oil and lower GP content in CN5, can justify the obtained results, also considering that the free GP phenolic compounds are more partitioned in the main water phase in CN16 and more active in the emulsion.

Figure S3 shows the Totox value for N5 and N16 samples and the respective controls during the accelerated test of oxidative stability. It is a comprehensive indicator used to evaluate the stability of food oils and is calculated from both peroxide and anisidine values. The results confirm those discussed above, being that the protective effect of the oxidation process was more evident for N16, with respect to the N5 sample.

The protective effect of GP towards oxidation has been reported by other authors, including Tseng and Zao (2013), who reported a 35–65% reduction of peroxide values in GP-added salad dressing during refrigerated storage for three weeks [14]. They added GP pomace, as dried whole powders, in lower amounts (1–3%) compared to this work, as well as pomace extracts, obtained by 70% acetone. In another study, the antioxidant properties of ethanolic extracts produced from the GP of different grape varieties were investigated in bulk soybean oil and oil-in-water emulsion systems [26]. The authors, after enriching the emulsions with GP extract at 400–1600 µg/g, subjected them to accelerated oxidation (convention oven at 50 °C), and found that the GP extract from red GP at the higher concentration was effective in inhibiting lipid oxidation.

**Figure 3:**
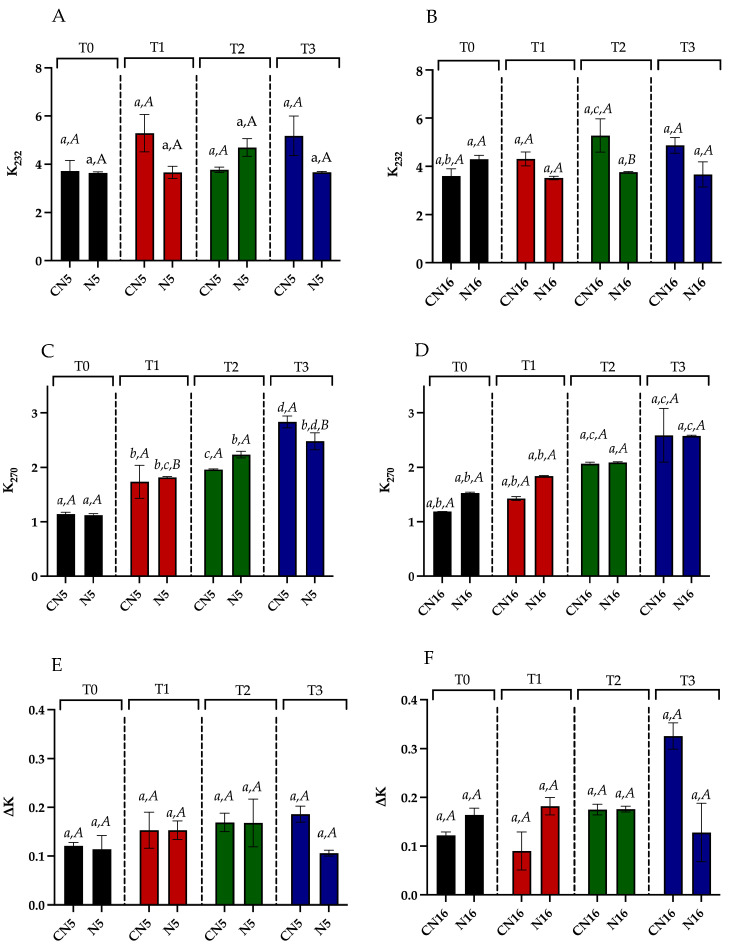
Values of K232 (**A**,**B**), K270 (**C**,**D**), and ΔK (**E**,**F**) for GP-added samples, N5 and N16, respectively, and the relative controls (CN5, CN16) at different storage times (T0, T1, T2, T3). Different uppercase letters indicate significant differences (*p* < 0.05) between the added and control sample at each time; different lowercase letters indicate significant differences (*p* < 0.05) between different times for each added and control sample. T0, immediately after their production; T1, after 7 days at 60 °C; T2, after 14 days at 60 °C; T3, after 21 days at 60 °C. N5 and N16 correspond to the samples reported in Table 1.

Figure 3A–F shows the results obtained for the spectrophotometric indexes in the N5 and N16 samples, and the relative controls. 

The K232, K270, and ΔΚ UV coefficients provide a good oxidative marker, characterizing the spectra of oxidized oils due to their consideration of both primary and secondary oxidation products [31]. In particular, during oxidation, conjugated dienes and trienes are formed, and they absorb 232 and 270 nm UV radiation, respectively. ΔK is an additional measure of the absorbance at 270 nm. The results for K232, reported in Figure 3A,B, show generally lower values for the N5 series, considering both the added and control samples, than the N16 series. Regarding the protective effect of GP, it can be observed in the N5 series at T1 and T3, and in the N16 series at T1, T2, and T3, even if significant differences were found only for N16 at T2. Regarding K270 results (Figure 3C,D), a slight increase was observed for both the N5 and N16 series during the accelerated test, and the protective effects of GP were detected only for the N5 samples at T3. Lastly, ΔK results highlighted the antioxidant capacity of phenolic compounds from GP only at the end of the accelerated test, as it possible to observe in Figure 3E,F. However, the lower ΔK values obtained at T3 for both N5 and N16 samples were not statistically different from the respective control.

To summarize, the effect of GP’s addition to the selected emulsions in delaying the oxidative progress was mainly highlighted by peroxide, anisidine, and Totox values, and it was more pronounced in the N16 sample.

The ability of phenolic compounds, extracted from GP and fractionated by preparative chromatography, to inhibit the oxidation of fish oil and its emulsion with water and frozen fish muscle, was studied [31]. The authors suggested that flavanol oligomers were potent inhibitors of oxidation in emulsions, and that monomeric flavanols were more effective than monomeric glycosylated flavonols. The rate of oxidation of polyunsaturated fatty acids was monitored by the formation of hydroperoxides and aldehydes, as well as of diene and triene conjugates. Other authors demonstrated the antioxidant activity of the complex bacterial cellulose and polyphenolic extract from GP by monitoring the decrease in conjugated dienes in olive oil-in-water emulsions [32]. Dordoni et al. investigated the effect of a grape skin extract addition on the oxidative stability of walnut paste [33]. They carried out an accelerated storage test on walnut paste, with or without an added antioxidant, for 15 days at 60 °C, simulating 2-year storage at 20 °C, based on an estimated activation energy of 80,327 kJ/mol for walnut lipid oxidation. By monitoring peroxides, conjugated dienes and trienes, total phenolics, ABTS, ORAC, FRAP, and tocopherols, they found that 5000 ppm extract addition did not prove to enhance the oxidative stability, nor the antioxidant properties of the walnut paste.

### 2.3. Determination of Antioxidant Properties and Phenolic Compounds

The next steps of the study involved the evaluation of the antioxidant activity by in vitro assays and the investigation of the qualitative and quantitative profile of phenolic compounds by HPLC-DAD. Table 3 shows the results of the spectrophotometric assays carried out to determine the total phenol content (TPC) and evaluate the antioxidant activity of the GP-added samples selected based on the experimental design, N5 and N16, at the different times of the accelerated stability test.

It is possible to observe that the total phenol content gradually decreased in both series, with a significant reduction from T0 to T1 of 73.7% in N5 and 44.2% in N16.

A significant reduction of TPC from T1 to T2 was also observed, while no differences were recorded from T2 to T3 (*p* > 0.05). At the final point, the total TPC reductions were 87.1 and 70.9% for N5 and N16, respectively. This loss of phenols must necessarily be attributed to the heat treatment. The stability of phenols is a very relevant issue, especially considering the growing evidence of their bioactivity. Unfortunately, phenols are not very stable compounds, as their chemical structure makes them sensitive to heat, pH variations, light, enzymatic activities, and the presence of metal ions and oxygen [34]. Temperature stands out among the factors that can affect the stability of phenolic compounds, since their molecular structure is thermolabile and can easily oxidize [35]. Moreover, it has been verified that the stability of phenolic compounds depends not only on its chemical nature, but also on the overall composition of the matrix, which could enhance or mitigate the degradation of specific compounds. Esparza and coworkers studied the stability of grape stem extracts during storage at 25 and 40 °C, in amber and transparent vials [35]. They found that the total polyphenols decreased significantly over time, and that samples stored at higher temperatures showed significantly lower polyphenol content.

Furthermore, the antioxidant activity of the N5–N9 samples was monitored during the accelerated test of oxidative stability. Three different spectrophotometric in vitro assays were used, two of which (DPPH and ABTS assays) are among the most popular to assess the antiradical properties, while the third (FRAP assay) is commonly carried out to measure the reducing capacity of pure compounds or mixtures. The results, reported in Table 3, show the expected reduction of the antioxidant activity of the samples. At T0, slightly higher values were observed for the three measurements in N16 compared to N5 (*p* < 0.05), probably due to some GP components not detected by the Folin–Ciocalteau assay. The DPPH value was markedly reduced already at T1 (N5, 87.3%; N16, 85.1%), as well as ABTS (N5, 94.3%; N16, 80.8%). FRAP values underwent a less marked reduction at T1 (N5, 60%; N16, 61.8%).

A good correlation was obtained between TPC and each antioxidant assay, confirming the accuracy of the analytical data. TPC correlated very well with ABTS (0.8430) and DPPH (0.7648), and less well with FRAP (0.4845), considering all eight samples in Table 3. The highest correlation, 0.9779, was observed between DPPH and ABTS values. Other authors reported that the antioxidant activity, measured by the DPPH assay for phenolic extracts, decreased as a function of temperature [35].

To better investigate the changes in the phenolic fraction during the accelerated oxidative stability test, the main GP phenols were characterized by HPLC-DAD analysis. Table 4 shows the concentration of phenolic compounds for the samples of the two N5 and N16 series. The results at T0 highlight that epicatechin was the most represented compound in both samples, followed by quercetin, catechin, procyanidin B2, and gallic acid in N5, and by peonydin-3-*O*-glucoside, quercetin, catechin, and gallic acid in N16. Other minor components have been determined in the two series samples. The differences observed between samples N5 and N16 at T0 deserve a comment first. Higher phenol concentrations were generally observed in the second compared to the first sample (*p* < 0.05); however, the quantitative profile of phenols can be attributed to the higher GP addition in N16 and the different water-to-oil ratio of the formulations (N5, 70% oil; N16, 60% water). For example, peonidin-3-*O*-glucoside was over ten times more concentrated in N16 than in N5, attributable to the high water solubility of this compound. Regarding the concentrations measured during the heat treatment, for some compounds, the expected decrease was observed, such as for procyanidin B2, ferulic acid, resveratrol, quercetin-3-*O*-glucoside, kampherol-3-*O*-glucoside, quercetin, and peonydin-3-*O*-glucoside. Interestingly, for epicatechin, an increased concentration has been measured at T1 compared to T0 for both N5 and N16 (*p* < 0.05).

It is widely reported that phenolic compounds are present in nature, both in free form and bound to polysaccharides and proteins. Notably, the phenolic compounds present in the bound form can far exceed the proportion of free compounds in many foods [36]. The results of a stability study of flavan-3-ols, epicatechin and catechin, and related dimeric procyanidins revealed that the dimers were less stable than monomers for both acidic and alkaline pH, and that incubation of dimer B2 and dimer B5 in acidic pH resulted in degradation to epicatechin [37]. Other authors have investigated the changes in the catechin and epicatechin content of grape seeds during storage under different water activity conditions [38]. In addition to the reduction of about 50% of the total phenol content at high humidity levels, they observed that catechin and epicatechin content were reduced during storage, particularly at 75% humidity. Epicatechin proved to be less sensitive to water activity conditions than catechin content. The same authors observed the continuous increase in gallic acid content and reported that it was a clear indication of hydrolytic processes in the system during storage. In this study, a slightly but significant higher content of gallic acid was measured at T1 with respect to T0 for N5. The formation of gallic acid is evident as a result of the hydrolysis of more complex compounds present in grape seeds (e.g., epicatechin gallate). Other interesting increasing trends were recorded for protocatechuic and p-coumaric acid from T0 to T2 in both N5 and N16 samples, and for caffeic and chlorogenic acid from T0 to T1.

These hydroxybenzoic and hydroxycinnamic acid derivatives have been reported to occur in bound form in plant matrices, forming, e.g., amides, esters, and glycosides [39].

The results reported in Table 4 show that the heat treatment in low pH conditions lead to the complete degradation of some phenolic compounds during the heat treatment. For example, peonidin-3-O-glucoside was found to be particularly unstable in formulates, and at T1 it was already no longer detected. In this regard, Sidani and Makris investigated the stability of red grape pomace anthocyanins in a juice model matrix (pH 3.48) [40]. They found that the treatment of the model solutions at 80 °C induced anthocyanin decomposition, which obeyed first order kinetics. Other authors reported that anthocyanins were significantly affected by temperature, and light enhanced their degradation when grape stem extracts were kept at 40 °C [35]. The low stability of grape phenolic compounds represents a challenge for researchers looking for methods to increase its stability. In this regard, the encapsulation approach has been widely investigated. Tsali and Goula, for example, investigated the storage stability of phenolic extract of non-encapsulated and encapsulated extracts from GP during storage at 60 °C, which is one of the temperatures recommended for accelerated shelf life studies [41]. GP extract, encapsulated by spray-drying using mixtures of maltodextrin and milk proteins as wall materials, underwent lower phenol degradation with respect to the crude extract.

The expected results of the stability study of phenolic compounds during the accelerated test indicated phenol low stability, as well as some interesting profile alteration during the heat treatment. It is worth noting that the accelerated test was carried out to evaluate the protection of GP-enriched dressing from oxidative progress, and that the proposed innovative formulations are not intended primarily for cooking treatment and require storage in refrigerated conditions.

### 2.4. Sensory Evaluation

**Figure 4:**
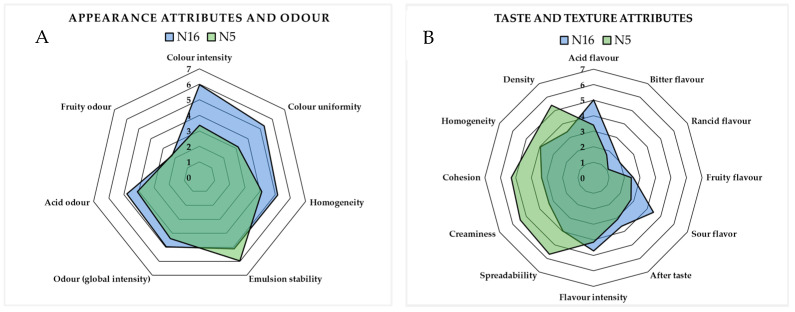
Sensory descriptive analysis: appearance attributes and odor (**A**); taste and texture attributes (**B**).

Sensory descriptive analysis of food products provides an understanding and control of the key attributes for consumer satisfaction and market success [42]. When developing innovative products by adding plant materials, such as agri-food waste, a sensory testing phase is highly recommended. The results of the sensory analysis for N5 and N16 samples are shown in Figure 4A,B. Regarding appearance attributes, the higher score for color intensity was obtained for N16 (6.0), with respect to the N5 sample (3.4), reflecting the higher GP addition. Moreover, the high water content and the low pH value are relevant factors influencing the color of anthocyanins. The color of anthocyanins is dependent on the pH of the solution, which is because of the molecular structure of anthocyanins having an ionic nature [43].

Figure 4A shows that N16 has a higher score for color uniformity and homogeneity than N5. Moreover, a slightly higher acid odor and odor (global intensity) were obtained for the N16 sample. The assessors assigned similar and very low scores for fruity odor, while a higher emulsion stability was observed for N5 compared to N16. Regarding the taste attributes, shown in Figure 4B, higher scores were obtained for acid and sour taste in N16 (4.8 and 4.5, respectively). This sample also resulted in slightly bitter and rancid tastes, which is different from N5. The fruity taste had a low and similar score for N5 and N16. A slightly higher aftertaste and flavor intensity was also recorded for N16. The opposite trend was obtained for the texture attributes; in fact, the assessors gave higher scores to N5 compared to N16 for all of the tested attributes, that is, density (5.4 vs. 3.4), homogeneity (4.7 vs. 4.0), cohesion (5.3 vs. 3.4), creaminess (5.5 vs. 3.3), and spreadability (5.7 vs. 4.0). Finally, the panelists were asked to rate the overall acceptability, and the results indicated that it was high for N5 (5.6), while it was lower for N16 (3.2). It should be noted that the GP addition is particularly high in the N16 sample in comparison to other studies. For example, other authors used 0.5–2% GP for the preparation of GP-enriched salad dressing, and the products with 0.5–1% were mostly liked by consumers based on the sensory study [14]. Several studies have reported a negative correlation between winery by-product concentrations and overall yogurt acceptance [14,23]. A significant decrease in overall acceptance, taste, odor, and appearance of yogurts with different added grape skin flours (6%) was recorded [23].

The results of sensory analysis obtained in this research indicate that the mayonnaise-like product N5 has the potential to develop as an innovative functional food with the zero-waste approach, as GP can be exploited without complex and expensive pre-treatment. However, the large-scale application of the proposed investigation requires a broader population study (involving individuals from different demographics and cultures), as well as addressing possible regulatory barriers pertaining to the use of GP as a food ingredient.

## 3. Materials and Methods

### 3.1. Materials, Reagents, Samples

Folin and Ciocalteu’s phenol reagent, 2,2′-azino-bis (3-ethylbenzothiazoline-6-sulphonic acid), diammonium salt (ABTS), 2,2-diphenyl-1-picrylhydrazyl (DPPH radical), gallic acid, (±)-6-hydroxy-2,5,7,8-tetramethylchromane-2-carboxylic acid (Trolox), methanol (MeOH), chloroform, acetic acid, sodium thiosulphate, protocatechuic acid, (+)-catechin, procyanidin B2, chlorogenic acid, caffeic acid, *p*-coumaric acid, ferulic acid, resveratrol, and quercetin were provided by Sigma-Aldrich (Milan, Italy). (-)-epicatechin, quercetin-3-*O*-glucoside, kampferol-3-*O*-glucoside, and petunidin-3-*O*-glucoside were provided by Extrasynthase (Genay, France). Cyclohexane was provided by VWR Chemicals (Milan, Italy). GP derived from the Merlot grape variety (certified by the producer) was supplied by Chiorri S.S., a local winery located in the province of Perugia (Central Italy). Its proximate composition is reported in Table S3. Commercial mayonnaise (a traditional egg-yolk mayo) and the ingredients (sunflower oil, vinegar, and lecithin) used for the formulation of the functional salad dressing were bought from a local market.

### 3.2. Development of GP-Based Dressing—Design of Experiments

MODDE 5.0 experimental design software (UMETRICS AB, Umeå, Sweden) was employed to evaluate the influence of four independent variables (water content, GP content, lecithin and vinegar content) on the viscosity and syneresis of the GP-added formulations. Table 5 shows the set values for the four factors.

The parameters selected as responses of the predictive model were the viscosity (expressed as mPa·s) and the syneresis of the final product, determined as reported in Section 2.3. A two-level full factorial experimental design, including three replicated center points (N17–N19), was employed. A total of 19 experiments were performed in a random order, following the conditions outlined in the worksheet (Table 1, Section 2.1). The data were fitted using a multiple linear regression (MLR) model.

The samples were prepared according to the experimental conditions indicated in the worksheet (Table 1, Section 2.1). In particular, water, GP, lecithin, and vinegar were mixed in a beaker and placed under gentle magnetic stirring until a homogenous mixture was obtained. Magnetic stirring was maintained for 24 h at room temperature. Then, the corresponding amount of sunflower oil was added drop by drop, while the mixture was mixed at 14,000 rpm with a homogenizer (SilentCrusher M model, Heidolph Instruments GmbH & Co. KG, Schwabach, Germany).

The N1–N19 samples were characterized as detailed in the following Section 3.3, Section 3.4 and Section 3.5.

### 3.3. Viscosity and Syneresis/Oil Separation

The viscosity of freshly produced GP-added samples was evaluated using a ViscoTM-895 digital viscometer (ATAGO, Tokyo, Japan) at room temperature. The instrument was equipped with an A1 RE-77104 spindle and viscosity measurements were performed with a spindle rotation of 200 rpm.

The syneresis/oil separation index for GP-added samples was determined by centrifuging 10 g of each sample for 10 min at 5000 rpm (Neya 8, REMI, Mumbai, India). After centrifugation, samples were visually evaluated to observe the possible separation of the water and oil phases. If present, water and/or oil phases were collected in a falcon tube and weighted. Syneresis/oil separation for each phase was calculated as follows:

$$Syneresis/Oil\;separation\left(\%\right)=\frac{Weight\;of\;separated\;water/oil\;phase(g)}{Initial\;weight\;of\;sample\;(g)}\times 100$$

### 3.4. pH Measurement

The pH measurement for GP-added samples (N1–N19) was performed on the aqueous phase recovered after the centrifugation process described in the previous paragraph, using a 334-B digital pH-meter (AMEL S.r.l, Milan, Italy).

### 3.5. Color Analysis

The color analysis of GP-mayo samples was evaluated by employing a benchtop tristimulus CLM-196 colorimeter (Eoptis, Trento, Italy). The results were expressed using the CIELAB scale, as follows: L* for lightness, a* for red/green coordinates, b* for blue/yellow coordinates. Furthermore, the Chroma (C*) parameter was calculated to evaluate color saturation. The color difference between samples was expressed using the following equation:

$$dE*ab=\sqrt{{{da*}^{2}{+db*}^{2}+dL*}^{2}}$$

where dE*ab is directly correlated with the greater difference between the two colors considered, allowing for the quantification of color diversity between the samples (GP-mayo) and the color of a commercial mayonnaise, chose as the reference color. The methodology employed in this paper has already been described in previous manuscripts [28,44].

### 3.6. Preparation of Optimized GP-Added Formulations

Based on the results obtained by the experimental design, two samples with higher viscosity and stability were selected, one with high oil content (mayonnaise-like product) and the other with high water content (salad dressing-like products). They were prepared as described in Section 3.2, together with the respective control samples, characterized by the same sample composition without a GP addition.

### 3.7. Accelerated Oxidative Stability Study

The evaluation of the oxidative stability of the selected high-oil and high-water GP-added emulsions was performed by an accelerated stability study. Four aliquots of each of the two GP-added samples, and of the respective controls, were prepared. Freshly produced samples were analyzed immediately after their production (Time 0, T0), while the other aliquots were maintained inside a forced convection laboratory oven (Binder FD53, Tuttlingen, Germany) at 60 °C. The heated samples were withdrawn every 7 days at three time points (T1, T2, and T3), over a total storage period of 21 days. The conditions for the accelerated test were adopted, since it has been reported that 60 °C for 15 days corresponds to 2 years at 20 °C [33]. For each time point of the stability study (T0, T1, T2, and T3), oil was recovered from the samples to assess the parameters of oxidative stability. Firstly, samples were frozen at −4 °C to facilitate the disruption of the emulsion. After 24 h, they were allowed to thaw at room temperature, and then they were centrifuged at 5000 rpm for 20 min at room temperature to obtain a full separation of the aqueous and oily phase. After centrifugation, the oily phase was collected and stored at −4 °C for subsequent analyses.

#### 3.7.1. Determination of Peroxide Value

Peroxide values for each sample were determined following a standard IOC procedure, based on a reaction with potassium iodide solution and a final titration with 0.01 N sodium thiosulphate [45]. The peroxide value of each sample, expressed in milliequivalents of active oxygen per kilogram (mEq O_2_/kg), is given by the following formula:

$$Peroxide\;value\;(PV):\frac{V\times N\times 1000}{W}$$

where V and N are, respectively, the volume and the normality of sodium thiosulphate used during titration, and W is the weight of the sample.

#### 3.7.2. Determination of Anisidine Value and Totox Value

The anisidine value was evaluated using a standard AOCS procedure [46] by a LAMBDA™ UV-Vis spectrophotometer (Perkin Elmer, Inc., Waltham, MA, USA). Briefly, the sample was dissolved in isooctane and absorbance (A_1_), and it was read at 350 nm against a blank (isooctane). Subsequently, the oil solution was combined in a reaction tube with p-anisidine solution (0.25% *w/v* in acetic acid). The solution was vortexed and placed in the dark. Absorbance (A_2_) was read after 10 min, against a solution composed of isooctane and p-anisidine, prepared using the same procedure. The anisidine value (AV) was determined using the following equation:

$$Anisidine\;value\;(AV)=\frac{25\times (1.2\;A_{2}-A_{1})}{W}$$

The Totox value, a comprehensive indicator of the overall oxidation of fats and oils, was calculated using the following equation:

TOTOX = 2 × PV + AV

#### 3.7.3. Determination of Spectrophotometric Indexes

The evaluation of oxidized compounds (conjugated dienes and trienes) in the samples was performed according to IOC procedure [47]. Of each oil, 50 mg was weighted in a 10 mL volumetric flask and brought to volume with cyclohexane. Solutions were swirled until reaching complete solubilization, and the absorbance was measured at 232 nm, 266 nm, 270 nm, and 274 nm using a LAMBDA™ UV-Vis spectrophotometer (Perkin Elmer, Inc., Waltham, MA, USA). The extinction coefficient values (K232 and K270) were calculated as follows:

$$K232\;or\;270=\frac{A232\;or\;A270}{C\times L}$$

where A232 and A270 are the absorbance values at the respective wavelengths (232 and 270 nm), C is the sample concentration (g/100 mL), and L is the optical path length (cm).

The extinction coefficient variation (∆K) was calculated according to the following equation:

$$\Delta K=K270-\frac{K266+K274}{2}$$

where K266, K270, and K274 are the extinction coefficient values obtained from the absorbance at the respective wavelengths.

### 3.8. Characterization of Phenolic Compounds and Antioxidant Activity

The samples obtained from the previous accelerated test of oxidative stability were also subjected to the evaluation of phenolic compounds and antioxidant properties, aiming to analyze them in the optimized formulations and monitor their changes during the heat treatment. The phenolic fraction was extracted from GP-added samples using a protocol described in a previous work [48], with few modifications. Briefly, a sample aliquot was added with MeOH and placed under magnetic stirring for 30 min at room temperature. After 30 min, the extraction mixture was centrifugated for 15 min at 5000 rpm. The supernatant was then recovered and stored at 4 °C for further spectrophotometric and chromatographic analyses.

#### 3.8.1. Evaluation of the Total Phenol Content and In Vitro Antioxidant Activity

The spectrophotometric analyses were employed to evaluate the total phenol content and the in vitro antioxidant activity of the two optimized GP-added formulations at the different time points of the accelerated test. All assays were performed according to previously described procedures [28,49,50].

TPC was evaluated by employing Folin and Ciocalteu’s phenol reagent and measuring the absorbance at 765 nm. The calibration curve obtained with gallic acid (y = 11.732x − 0.0825; R^2^ > 0.999) was employed for quantification, and the results were expressed as mg of gallic acid equivalents per gram of GP-added sample (mg GAE/g). The in vitro antioxidant activity of the extracts was evaluated by investigating both the free radical scavenging activity (ABTS and DPPH assays) and the reducing capacity (ferric reducing antioxidant power, FRAP assay).

For ABTS and DPPH assays, the absorbance was measured at 734 and 517 nm, respectively. For the FRAP assay, the absorbance was measured at 593 nm. Trolox was used as the standard to obtain calibration curves for the ABTS (y = −1.6595x + 0.6945), DPPH (y = −1.4522x + 0.6126), and FRAP (y = 9.483x − 0.0504) assays. Each calibration curve had good linearity (R^2^ > 0.999). The results for the ABTS, DPPH, and FRAP assays were reported as mg of Trolox equivalent per gram of GP-added sample (mg TE/g).

#### 3.8.2. HPLC-DAD Analysis

Qualitative and quantitative analysis of the phenolic profile was performed by employing a Waters^®^ Acquity Arc HPLC system (Waters Corporation, Milford, MA, USA) equipped with a low-pressure mixing quaternary solvent manager-R (QSM-R) as a high-pressure pump, a sample manager flow through needle-R (FTN-R) using a direct-injection mechanism, a column heather/cooler, and a Waters 2998 photodiode array (PDA) detector. Waters^®^ Empower 3^®^ Chromatography Data Software (CDS) was employed for data management. Phenolic compound separation and quantification were performed on a X-Bridge (150 × 4.6 mm, 5 µm) column. Two mobile phases were employed, as follows: A (water/formic acid, 0.1% *v*/*v*) and B (MeOH), using the following gradient, 0 min—100% A, 21 min—70% A, 27 min—60% A, 33 min—40% A, 40 min—100% A. The flow rate was 0.7 mL/min and the column temperature was set at 25 °C.

Before injection, methanolic extracts were filtered with a 0.22 µm nylon syringe filter (ClearLine^®^, Bernolsheim, France) and collected in hermetically sealed Eppendorf ™.

The quantification of the different phenolic compounds was performed by employing calibration curves obtained from the external standard corresponding to the individual identified compounds. Protocatechuic acid was evaluated at 260 nm and gallic acid at 270 nm, while (+)-catechin, (−)-epicatechin, and procyanidin B2 were evaluated at 278 nm. Chlorogenic, caffeic, p-coumaric, ferulic acids, and resveratrol were quantified at 320 nm. Quercetin-3-*O*-glucoside, kampferol-3-*O*-glucoside, and quercetin were quantified using 360 nm as wavelength, and petunidin-3-*O*-glucoside was quantified at 528 nm. The validation parameters have been reported in a previous paper [49].

### 3.9. Sensory Analysis

A sensory evaluation of the N5 and N16 samples was also performed. A total of 20 trained assessors, aged between 22 and 59 years, participated in the panel test. The tasters were asked to evaluate the two GP-added formulations in terms of visual and olfactory assessment (color intensity, color uniformity, homogeneity, emulsion stability, odor—global intensity, acidic odor, fruity odor) and in terms of flavor and texture characteristics (salty flavor, acid flavor, bitter flavor, rancid flavor, fruity flavor, metallic flavor, sour flavor, aftertaste, flavor intensity, spreadability, creaminess, cohesion, homogeneity, density). Furthermore, the assessors were also asked to give a comprehensive evaluation of the product in terms of overall acceptability.

Each assessor received sheets with a seven-point scale for each attribute, with one indicating the lowest intensity and seven indicating the highest intensity. Evaluations were performed in individual booths, following the ISO 4121:2003 criteria [51]. The samples were served to the tasters on plastic plates with spoons at room temperature. Each taster was provided with water to cleanse their palate between tasting different samples. Individual scores of each panel were elaborated to obtain an average value, used as a score for the taste panel. For each GP-mayonnaise sample, three evaluations were performed.

### 3.10. Statistical Analysis

All analyses were performed in triplicate (n = 3), with results expressed as the mean ± standard deviation (SD). Statistical significance was assessed using one-way analysis of variance (ANOVA), followed by Tukey’s HSD (honestly significant difference) post hoc test. A *p*-value less than 0.05 was considered statistically significant. GraphPad PRISM version 9.3.1 (GraphPad Software, Boston, MA, USA) and Excel Version 16.91 (Microsoft, Redmond, WA, USA) for Windows were used for statistical analysis and graph generation.

## 4. Conclusions

There is increasing interest in using fruit processing wastes as functional food ingredients, since they are a rich source of dietary fiber and bioactive antioxidant compounds. GP represents an underutilized resource with immense potential to address both environmental and nutritional challenges. Its transformation into functional foods not only promotes sustainability, but also introduces health-promoting bioactive components into diets. In the present work, GP was used to improve the technological and nutritional/healthy properties of mayonnaise-like products and salad dressings. The experimental design approach, adopted to assess the impact of compositional factors on the viscosity and stability of the GP-added formulations, allowed the selection of two optimized samples with a different water–oil ratio. For this reason, they could have an interesting application in the development of different healthy salad dressings, with all of the advantages related to the full exploitation of GP, based on the zero-waste approach. The improved oxidative stability and syneresis reduction achieved by adding GP could be leveraged on an industrial scale to improve the emulsion properties and antioxidant activity of food formulations. The investigation also has interesting implications for sustainability and cost-effectiveness, as GP is a widely produced agri-food waste, entirely exploited for the development of healthy dressing, based on the zero-waste approach.

The deep characterization of the GP-added formulations allowed us to confirm the GP antioxidant activity, which was dependent on the amount added. Interesting results were obtained by the chromatographic characterization of phenolic compounds. Their quantitative profile was also dependent on the formulation composition, and in particular, the water–oil ratio. The sensory analysis results showed a high acceptability for the oil-rich formulation, while suggesting to further optimize the water-rich formulation, either by GP encapsulation or an additive addition, to improve both technological and sensorial properties.

Lastly, further research is necessary to face the actual challenges related to industrial implementation, considering the technological aspects, as well as the consumer acceptability and the regulatory aspects. Possible legal barriers pertaining to the use of GP as a food ingredient for mayonnaise production should be considered before moving from a laboratory scale to an industrial production scale.

## Figures and Tables

**Table 1:** Worksheet with the experimental conditions for the N1–N19 samples and relative responses.

	Factors				Responses	
Samples	Water (%)	GP (% *w*/*v*)	Lecithin (% *w*/*v*)	Vinegar (% *v*/*v*)	Viscosity (mPa∙s)	Syneresis (%Water)
N1	30	4	1	3	225.0	15.1
N2	60	4	1	3	71.8	32.4
N3	30	8	1	3	197.5	9.8
N4	60	8	1	3	203.8	20.1
N5	30	4	4	3	3193.6	0.0
N6	60	4	4	3	212.8	20.2
N7	30	8	4	3	2587.2	0.0
N8	60	8	4	3	122.3	22.3
N9	30	4	1	6	120.2	20.7
N10	60	4	1	6	35.6	41.5
N11	30	8	1	6	320.2	14.7
N12	60	8	1	6	211.4	37.4
N13	30	4	4	6	2065.3	5.3
N14	60	4	4	6	181.2	14.2
N15	30	8	4	6	620.0	1.3
N16	60	8	4	6	245.4	19.7
N17	45	6	2.5	4.5	306.7	19.7
N18	45	6	2.5	4.5	300.5	20.9
N19	45	6	2.5	4.5	291.3	22.1

**Table 2:** Results of the colorimetric analysis of the CM and N1–N19 samples.

Sample	L*	a*	b*	
CM	81.52 ± 0.03	−3.49 ± 0.01	11.69 ± 0.04	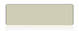
N1	39.41 ± 0.08	17.69 ± 0.04	2.90 ± 0.02	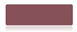
N2	39.33 ± 0.0	17.61 ± 0.05	4.17 ± 0.01	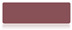
N3	15.20 ± 0.01	24.62 ± 0.01	−2.07 ± 0.02	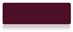
N4	28.81 ± 0.0	19.56 ± 0.01	2.06 ± 0.02	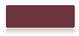
N5	27.99 ± 0.01	17.11 ± 0.04	1.67 ± 0.01	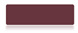
N6	52.99 ± 0.01	12.91 ± 0.01	2.17 ± 0.03	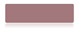
N7	23.28 ± 0.01	17.86 ± 0.04	0.85 ± 0.01	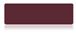
N8	46.09 ± 0.01	13.73 ± 0.01	2.54 ± 0.02	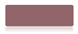
N9	24.52 ± 0.03	19.65 ± 0.02	1.81 ± 0.02	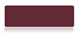
N10	26.22 ± 0.01	18.73 ± 0.02	3.08 ± 0.03	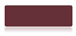
N11	16.86 ± 0.02	20.18 ± 0.07	0.83 ± 0.03	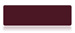
N12	20.77 ± 0.01	20.00 ± 0.02	1.72 ± 0.02	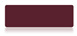
N13	40.73 ± 0.02	16.50 ± 0.02	2.61 ± 0.02	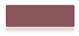
N14	53.80 ± 0.02	13.45 ± 0.02	2.71 ± 0.03	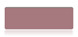
N15	16.78 ± 0.02	23.28 ± 0.02	−0.67 ± 0.02	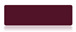
N16	35.49 ± 0.01	19.48 ± 0.03	2.76 ± 0.02	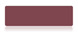
N17	45.95 ± 0.02	15.32 ± 0.01	1.09 ± 0.02	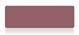
N18	44.97 ± 0.01	15.81 ± 0.01	1.44 ± 0.02	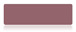
N19	45.07 ± 0.01	16.44 ± 0.01	1.06 ± 0.03	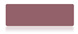

Results are expressed as means ± standard deviation (n = 3); CM, commercial mayonnaise; L*, Lightness; a*, green–red opponent colors; b*, blue–yellow opponent colors.

**Table 3:** Total phenol content and antioxidant activity (ABTS, DPPH, and FRAP) of N5 and N16 samples at different times of the accelerated oxidative stability test.

Samples	TPC (mg GAE/g)	DPPH (mg TE/g)	ABTS (mg TE/g)	FRAP (mg TE/g)
N5-T0	0.93 ± 0.03 ^a^	0.63 ± 0.03 ^a^	0.70 ± 0.02 ^a^	0.15 ± 0.01 ^a^
N5-T1	0.25 ± 0.02 ^b^	0.08 ± 0.006 ^b^	0.04 ± 0.00 ^b^	0.06 ± 0.00 ^b^
N5-T2	0.15 ± 0.02 ^c^	0.09 ± 0.005 ^b^	-	0.02 ± 0.00 ^c^
N5-T3	0.12 ± 0.01 ^c^	0.06 ± 0.005 ^b^	-	0.17 ± 0.00 ^d^
N16-T0	0.86 ± 0.02 ^a^	1.14 ± 0.05 ^a^	1.20 ± 0.02 ^a^	0.34 ± 0.00 ^a^
N16-T1	0.48 ± 0.01 ^b^	0.17 ± 0.00 ^b^	0.23 ± 0.01 ^b^	0.13 ± 0.00 ^b^
N16-T2	0.24 ± 0.01 ^c^	0.14 ± 0.00 ^b^	0.08 ± 0.01 ^c^	0.06 ± 0.00 ^c^
N16-T3	0.25 ± 0.01 ^c^	0.16 ± 0.02 ^b^	-	0.06 ± 0.00 ^c^

TPC, total phenol content; GAE, gallic acid equivalents; ABTS, 2,2′-azino-bis(3-ethylbenzothiazoline-6-sulfonic acid) diammonium salt; DPPH, 2,2-diphenyl-1-picrylhydrazyl; FRAP, ferric reducing antioxidant power; TE, Trolox equivalents. -, not determined. Results are expressed as mean ± standard deviation (n = 3) on weight of GP mayonnaise sample. Different letters in each column indicate significant differences with *p* < 0.05 between samples of each of the two series. T0, immediately after their production; T1, after 7 days at 60 °C; T2, after 14 days at 60 °C; T3, after 21 days at 60 °C. N5 and N16 correspond to the samples reported in Table 1.

**Table 4:** Concentration (mg/g) of phenolic compounds in N5 and N16 samples at different times of accelerated oxidative stability test (mean values ± SD, *n* = 3).

	N5			
	T0	T1	T2	T3
Gallic acid	2.18 ± 0.17 ^a^	2.63 ± 0.00 ^b^	1.12 ± 0.05 ^c^	0.72 ± 0.04 ^c^
Protocatechuic acid	<LOD	4.91 ± 0.07 ^a^	5.41 ± 0.11 ^b^	3.20 ± 0.01 ^c^
Catechin	6.1 ± 0.39 ^a^	1.35 ± 0.07 ^b^	6.38 ± 0.02 ^a,c^	0.97 ± 0.03 ^b^
Epicatechin	13.69 ± 0.14 ^a^	35.59 ± 0.20 ^b^	31.47 ± 0.81 ^c^	21.13 ± 0.24 ^d^
Procyanidin B2	3.01 ± 0.11 ^a^	0.54 ± 0.02 ^b^	0.50 ± 0.03 ^b^	0.54 ± 0.01 ^b^
Chlorogenic acid	0.17 ± 0.01 ^a^	0.27 ± 0.00 ^b^	<LOD	<LOD
Caffeic acid	1.64 ± 0.14 ^a^	2.14 ± 0.07 ^b^	1.05 ± 0.06 ^c^	0.68 ± 0.02 ^c^
*p*-Coumaric acid	0.27 ± 0.00 ^a^	0.44 ± 0.03 ^b^	0.51 ± 0.03 ^b^	0.26 ± 0.02 ^a^
Ferulic acid	0.34 ± 0.02	<LOD	<LOD	<LOD
Resveratrol	0.32 ± 0.00 ^a^	<LOD	<LOD	0.29 ± 0.00 ^b^
Quercetin-3-*O*-Glucoside	1.40 ± 0.04 ^a^	0.37 ± 0.01 ^b^	0.38 ± 0.04 ^b^	0.34 ± 0.02 ^b^
Kaempherol-3-*O*-Glucoside	0.69 ± 0.02 ^a^	0.24 ± 0.01 ^b^	<LOD	<LOD
Quercetin	7.50 ± 0.16 ^a^	1.85 ± 0.02 ^b^	0.82 ± 0.01 ^c^	<LOD
Peonidin-3-*O*-Glucoside	1.61 ± 0.03	<LOD	<LOD	<LOD
	N16			
	T0	T1	T2	T3
Gallic acid	3.63 ± 0.19 ^a^	3.23 ± 0.2 ^a^	0.71 ± 0.01 ^b^	1.46 ± 0.10 ^c^
Protocatechuic acid	0.22 ± 0.01 ^a^	10.48 ± 0.3 ^b^	20.96 ± 1.09 ^c^	13.69 ± 0.35 ^b^
Catechin	13.95 ± 0.16 ^a^	2.65 ± 0.20 ^b^	6.93 ± 0.34 ^c^	3.96 ± 0.12 ^d^
Epicatechin	21.67 ± 0.74 ^a^	71.20 ± 0.15 ^b^	59.82 ± 1.00 ^c^	26.94 ± 0.03 ^d^
Procyanidin B2	2.75 ± 0.07 ^a^	0.39 ± 0.01 ^b^	<LOD	<LOD
Chlorogenic acid	0.24 ± 0.00 ^a^	0.27 ± 0.01 ^a^	0.18 ± 0.01 ^a^	<LOD
Caffeic acid	2.73 ± 0.14 ^a^	4.94 ± 0.12 ^b^	4.02 ± 0.19 ^c^	1.02 ± 0.00 ^d^
*p*-Coumaric acid	0.32 ± 0.00 ^a^	0.72 ± 0.05 ^b^	0.70 ± 0.05 ^b^	0.39 ± 0.00 ^a^
Ferulic acid	0.40 ± 0.00 ^a^	0.19 ± 0.01 ^b^	<LOD	<LOD
Resveratrol	0.44 ± 0.02 ^a^	0.30 ± 0.01 ^b^	<LOD	<LOD
Quercetin-3-*O*-Glucoside	2.23 ± 0.08 ^a^	0.57 ± 0.02 ^b^	0.52 ± 0.03 ^b^	0.52 ± 0.02 ^b^
Kaempherol-3-*O*-Glucoside	1.31 ± 0.00 ^a^	0.46 ± 0.01 ^b^	0.16 ± 0.01 ^c^	<LOD
Quercetin	14.19 ± 0.19 ^a^	1.15 ± 0.02 ^b^	0.63 ± 0.01 ^c^	0.65 ± 0.02 ^c^
Peonidin-3-*O*-Glucoside	21.23 ± 0.21	<LOD	<LOD	<LOD

LOD, limit of detection. Different letters in each row indicate significant differences with *p* < 0.05. T0, immediately after their production; T1, after 7 days at 60 °C; T2, after 14 days at 60 °C; T3, after 21 days at 60 °C.

**Table 5:** Independent variables set for experimental design.

Independent Variables	Unit	Type	Setting
Water	% *v/v*	Quantitative	From 30 to 60
GP	% *w/v*	Quantitative	From 4 to 8
Lecithin	% *w/v*	Quantitative	From 1 to 4
Vinegar	% *v/v*	Quantitative	From 3 to 6

## Data Availability

Data are contained within the article.

## Supplementary Materials

The following supporting information can be downloaded at: https://www.mdpi.com/article/10.3390/molecules30030693/s1, Figure S1. Response prediction plots of the four investigated factors (A: water %; B: GP %; C: lecithin %; D: vinegar %) on viscosity and surface contour plot (E) for variables of water and GP percentages (with lecithin and vinegar content at the constant value of the center point); Figure S2. Response prediction plots of the four investigated factors (A: water %; B: GP %; C: lecithin %; D: vinegar %) on syneresis and surface contour plot (E) for variables of water and GP percentages (with lecithin and vinegar content at the constant value of the center point); Figure S3. Values of Totox for GP-added samples, N5 and N16 (A–B, respectively), and the relative controls (CN5, CN16), at different storage times (T0, T1, T2, T3). Different uppercase letters indicate significant differences (*p* < 0.05) between the added and control sample at each time, while different lowercase letters indicate significant differences (*p* < 0.05) between different times for each added and control sample; Table S1. Results of emulsion stability, measured as oil phase separation after centrifugation, and pH for the N1–N19 samples; Table S2. Colorimetric analysis. Differences between the color parameters of GP-added formulations and commercial mayonnaise; Table S3. Proximate composition of GP.
