# Supplementary material for: Harnessing Grape Pomace, a Multifunctional By-Product from the Wine Industry for High-Value Salad Dressings
Source: Molecules. 2025 Feb 5;30(3):693. doi: 10.3390/molecules30030693 (PMC11821133; doi:10.3390/molecules30030693)
Supplement: Supplementary file 1 [file molecules-30-00693-s001.zip › molecules-3405776-supplementary.pdf]

Harnessing grape pomace, a multifunctional by-product from the wine industry, for high-value salad dressings

Luciano Mangiapelo †, Nicola Pinna †, Francesca Blasi, Federica Ianni, Giuseppa Verducci and Lina Cossignani *

Department of Pharmaceutical Sciences, University of Perugia, 06126 Perugia, Italy; luciano.mangiapelo@dottorandi.unipg.it (L.M.); nicola.pinna@dottorandi.unipg.it (N.P.); francesca.blasi@unipg.it (F.B.); federica.ianni@unipg.it (F.I.); giuseppa.verducci@unipg.it (G.V.)

* Correspondence: lina.cossignani@unipg.it (L.C.); Tel.: +39-075-585-7959

† These authors contributed equally to this work

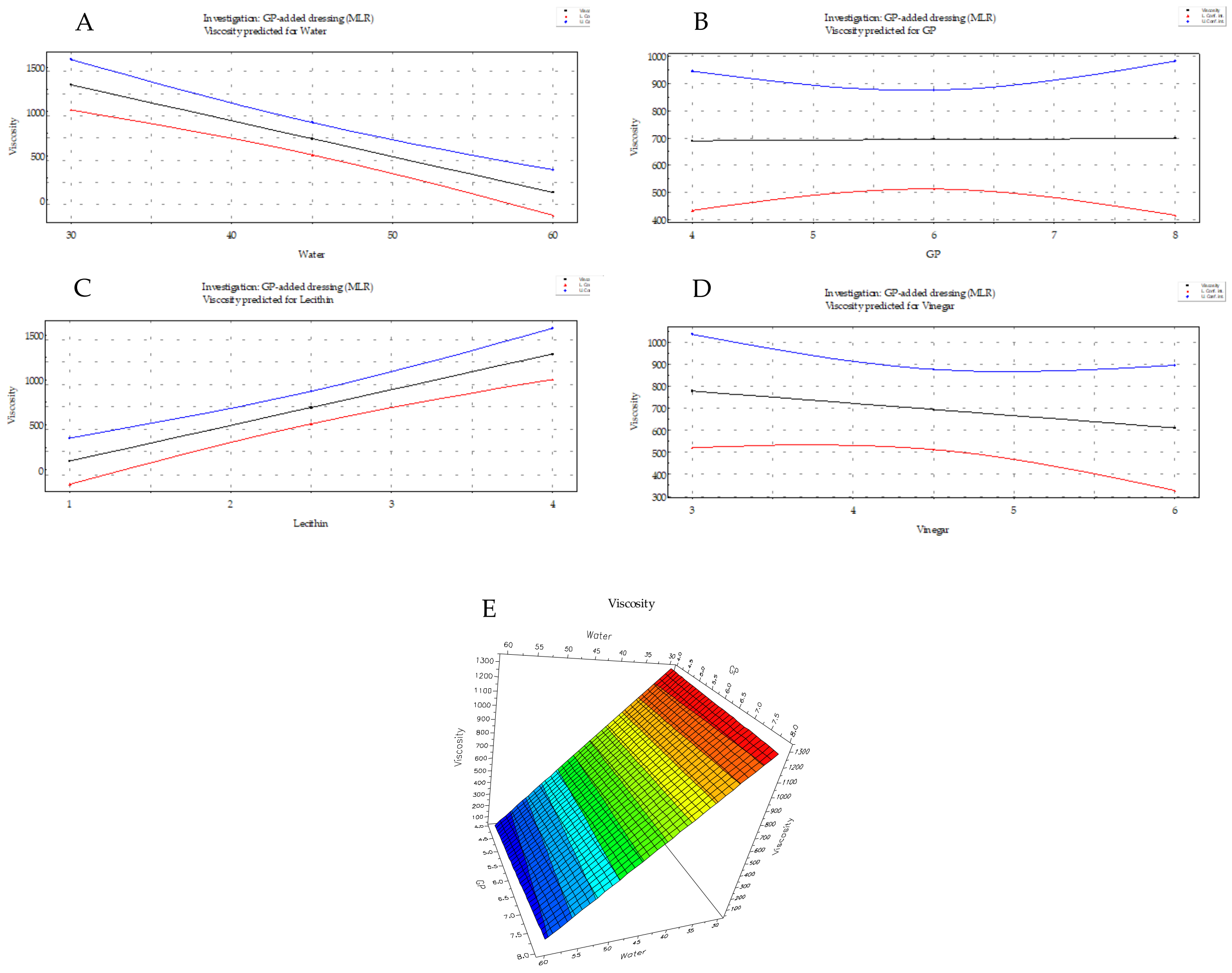

Figure S1. Response prediction plots of the four investigated factors (A: water %; B: GP %; C: lecithin %; D: vinegar %) on viscosity and surface contour plot (E) for variables water and GP percentages (with lecithin and vinegar content at the constant value of the center point).

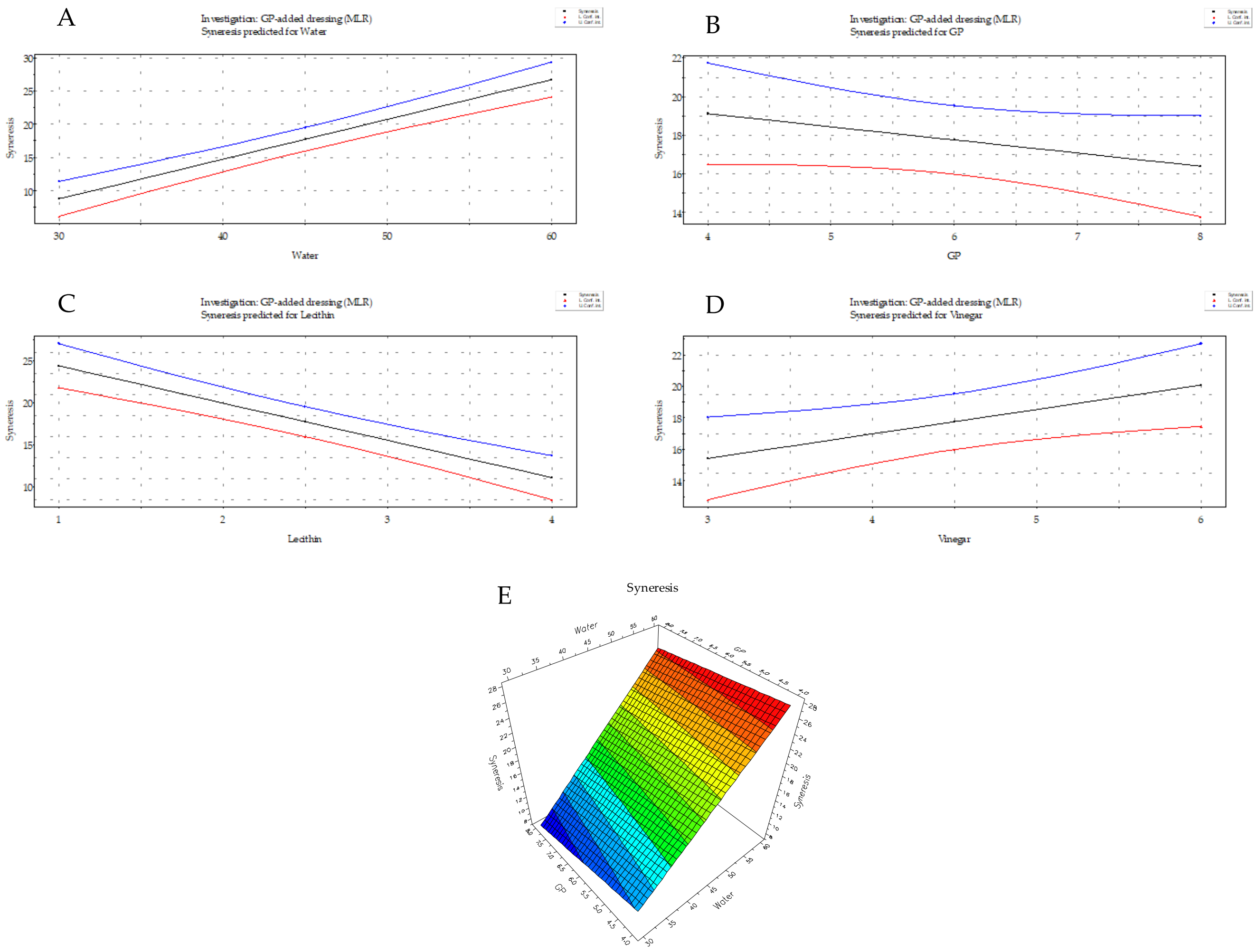

Figure S2. Response prediction plots of the four investigated factors (A: water %; B: GP %; C: lecithin %; D: vinegar %) on syneresis and surface contour plot (E) for variables water and GP percentages (with lecithin and vinegar content at the constant value of the center point).

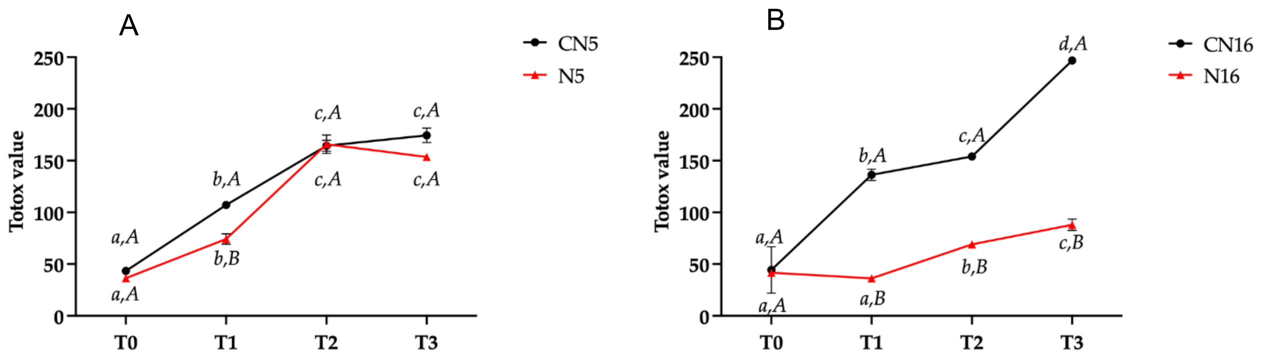

Figure S3. Values of Totox for GP-added samples, N5 and N16 (A-B, respectively), and the relative controls (CN5, CN16), at different storage times (T0, T1, T2, T3). Different upper-case letters indicate significant differences ($p < 0.05$) between added and control sample at each time, different lowercase letters indicate significant differences ($p < 0.05$) between different times for each added and control sample.

Table S1. Results of emulsion stability, measured as oil phase separation after centrifugation, and pH for the N1-N19 samples.

Sample	Oil separation (%)	pH
N1	46.9	3.38
N2	18.9	3.40
N3	56.2	3.72
N4	20.3	3.47
N5	54.1	3.40
N6	0.0	3.77
N7	45.3	4.25
N8	0.0	3.80
N9	53.5	3.82
N10	21.4	3.81
N11	48.4	3.90
N12	16.9	3.85
N13	42.0	3.81
N14	0.0	3.66
N15	56.6	3.61
N16	0.0	3.61
N17	0.0	3.48
N18	0.0	3.56
N19	24.4	3.54

Table S2. Colorimetric analysis. Differences between the colour parameters of GP-added formulations and commercial mayonnaise.

Sample N°	dE*ab	dL*	da*	db*	dC*
**N1**	47.92 ± 0.09	-42.08 ± 0.08	21.18 ± 0.03	-8.78 ± 0.02	5.74 ± 0.03
**N2**	47.74 ± 0.02	-42.16 ± 0.0	21.10 ± 0.04	-7.51 ± 0.01	5.91 ± 0.05
**N3**	73.31 ± 0.02	-66.29 ± 0.0	28.11 ± 0.06	-13.75 ± 0.02	12.51 ± 0.06
**N4**	58.34 ± 0.0	-52.68 ± 0.0	23.15 ± 0.01	-9.61 ± 0.01	7.57 ± 0.0
**N5**	58.21 ± 0.01	-53.51 ± 0.02	20.61 ± 0.05	-10.00 ± 0.02	5.00 ± 0.05
**N6**	34.23 ± 0.02	-28.21 ± 0.01	16.41 ± 0.01	-9.53 ± 0.03	0.9 ± 0.01
**N7**	62.94 ± 0.01	-58.21 ± 0.01	21.36 ± 0.01	-10.83 ± 0.0	5.70 ± 0.04
**N8**	40.42 ± 0.02	-35.40 ± 0.01	17.22 ± 0.01	-9.14 ± 0.02	1.77 ± 0.01
**N9**	62.55 ± 0.03	-57.25 ± 0.03	23.14 ± 0.02	-9.87 ± 0.02	7.53 ± 0.02
**N10**	60.19 ± 0.02	-55.28 ± 0.02	22.23 ± 0.01	-8.59 ± 0.03	6.79 ± 0.02
**N11**	69.67 ± 0.04	-64.63 ± 0.02	23.68 ± 0.06	-10.85 ± 0.02	8.01 ± 0.06
**N12**	65.87 ± 0.01	-60.72 ± 0.01	23.49 ± 0.02	-9.97 ± 0.02	7.89 ± 0.01
**N13**	46.29 ± 0.02	-40.75 ± 0.02	19.93 ± 0.02	-9.07 ± 0.02	4.52 ± 0.02
**N14**	33.83 ± 0.02	-27.69 ± 0.02	16.94 ± 0.02	-9.50 ± 0.03	1.43 ± 0.02
**N15**	71.12 ± 0.03	-64.71 ± 0.02	26.78 ± 0.03	-12.35 ± 0.02	11.10 ± 0.02
**N16**	52.18 ± 0.02	-46.01 ± 0.01	22.97 ± 0.04	-8.91 ± 0.02	7.49 ± 0.04
**N17**	41.58 ± 0.03	-35.55 ± 0.02	18.82 ± 0.01	-10.58 ± 0.02	3.18 ± 0.01
**N18**	42.56 ± 0.02	-36.52 ± 0.01	19.31 ± 0.01	-10.24 ± 0.02	3.69 ± 0.01
**N19**	42.85 ± 0.02	-36.41 ± 0.01	19.93 ± 0.01	-10.62 ± 0.03	4.28 ± 0.01

Table S3. Proximate composition of GP.

Total Dietary Fiber (g)	60.12 ± 0.26
- *Insoluble Fiber*	55.30 ± 0.11
- *Soluble Fiber*	4.82 ± 0.15
Fats (g)	11.12 ± 0.32
Carbohydrates (g)	4.18 ± 0.07
Protein (g)	11.07 ± 0.26
Moisture (g)	4.77 ± 0.02
Ash (g)	6.67 ± 0.02
